# Synthetic Genetic Interactions Reveal a Dense and Cryptic Regulatory Network of Small Noncoding RNAs in Escherichia coli

**DOI:** 10.1128/mbio.01225-22

**Published:** 2022-08-03

**Authors:** Kenneth Rachwalski, Michael J. Ellis, Madeline Tong, Eric D. Brown

**Affiliations:** a Institute of Infectious Disease Research, McMaster University, Hamilton, Ontario, Canada; b Biochemistry and Biomedical Sciences, McMaster University, Hamilton, Ontario, Canada; National Cancer Institute

**Keywords:** systems biology, bacterial carbon metabolism, small noncoding RNA

## Abstract

Over the past 20 years, we have learned that bacterial small noncoding RNAs (sRNAs) can rapidly effect changes in gene expression in response to stress. However, the broader role and impact of sRNA-mediated regulation in promoting bacterial survival has remained elusive. Indeed, there are few examples where disruption of sRNA-mediated gene regulation results in a discernible change in bacterial growth or survival. The lack of phenotypes attributable to loss of sRNA function suggests that either sRNAs are wholly dispensable or functional redundancies mask the impact of deleting a single sRNA. We investigated synthetic genetic interactions among sRNA genes in Escherichia coli by constructing pairwise deletions in 54 genes, including 52 sRNAs. Some 1,373 double deletion strains were studied for growth defects under 32 different nutrient stress conditions and revealed 1,131 genetic interactions. In one example, we identified a profound synthetic lethal interaction between ArcZ and CsrC when E. coli was grown on pyruvate, lactate, oxaloacetate, or d-/l-alanine, and we provide evidence that the expression of *ppsA* is dysregulated in the double deletion background, causing the conditionally lethal phenotype. This work employs a unique platform for studying sRNA-mediated gene regulation and sheds new light on the genetic network of sRNAs that underpins bacterial growth.

## INTRODUCTION

The model Gram-negative bacterium Escherichia coli K-12 has approximately 4,300 genes, only 303 of which are classified as being essential for growth under optimal nutrient conditions (1–3). However, gene essentiality is context dependent (4), wherein both the environmental (5–7) and genetic (8–12) background of the bacterium can alter the dispensability of genes. For example, when E. coli is grown on M9 minimal medium with glucose, an additional 119 genes become required for growth; these genes are largely involved in amino acid, vitamin, and nucleobase synthesis (2). Indeed, previous genome-scale investigations of conditional gene essentiality have found that nearly half of all genes in E. coli become required for normal growth under some condition (6, 13, 14). While the remaining ~50% of genes in E. coli may appear to be entirely dispensable for growth based on these screens, some growth phenotypes may be masked by genetic interactions that occur because of functional redundancy. For example, some nutrient transporters are dispensable for growth in both nutrient-rich and nutrient-poor growth media but become essential when genes encoding the cognate biosynthetic enzymes are disrupted (15). High-throughput synthetic genetic interaction studies enable genome-wide investigations of genetic backgrounds in which a dispensable gene becomes important for growth, often uncovering functional relationships between genes (16, 17). In this approach, a gene deletion of interest is systematically introduced into a library of defined gene mutants (2), yielding an array of unique double mutants that can be screened for phenotypes of interest, most often bacterial growth (12, 15, 18–20). Such studies provide unique information about mechanisms underpinning bacterial survival in diverse environments, as well as insights into gene function.

Previous genome-wide approaches directed at understanding gene function have focused primarily on protein-coding regions of the genome, overlooking noncoding genes, such as small regulatory RNAs (sRNAs). Where defined sRNA mutants have been included in screens for bacterial growth (6, 14, 18, 21) or represented in transposon sequencing (Tn-Seq) studies (13), there are few examples of profound growth phenotypes associated with disruption of sRNA genes. Targeted approaches to uncovering growth phenotypes for sRNA deletion strains have also been largely unsuccessful (22); in one example, ~1,900 conditions, including metabolic and chemical stressors, were screened without success to identify hypersensitivities of an E. coli Δ*sdsR* strain (23). This is perhaps paradoxical, as sRNAs are purported to be critical to bacterial stress responses (24, 25). In contrast, the major sRNA chaperone protein Hfq is required for survival under a variety of stress conditions (6, 26), including those caused by antibiotics that span multiple chemical and mechanistic classes (27). Furthermore, deletions in some 25 genes leading to nutrient stress were found to have synthetic sick/lethal interactions with the *Δhfq* mutation in E. coli (15). Although examples exist of Hfq acting independently of sRNAs to control mRNA translation (28–32), the best-defined role for this protein is as a chaperone for the action of sRNAs. Hfq binds and stabilizes client sRNAs, forms ternary complexes with sRNAs and mRNAs to increase local concentrations and promote correct orientation, and can play an active role in RNA restructuring to catalyze base-pairing interactions (33–35). Although most sRNAs enact their regulatory function by base pairing with target mRNAs, some sRNAs can function independently of Hfq and/or by binding to targets at the protein rather than the mRNA level (22, 36–38). The loss of functional Hfq abolishes almost all Hfq-dependent sRNA-mediated regulation (39), and the many phenotypes associated with an *hfq* disruption suggest that sRNAs as a whole are indeed important for bacterial survival. While many sRNAs depend entirely on Hfq’s chaperone activity, a number of sRNAs have been found to interact exclusively with other RNA-binding proteins, such as ProQ, and some 30% of sRNAs interact with multiple RNA chaperones (22, 25, 40–42). However, very few phenotypes have been uncovered for a ProQ deletion strain in E. coli (6, 14, 15, 18, 27), suggesting a lesser dependence on this chaperone than on Hfq for bacterial growth and survival.

Our understanding of sRNA-mediated regulation has evolved from the view where a single sRNA, in concert with a chaperone protein, can modulate the stability and/or translation of an mRNA to one where gene expression can be fine tuned through the parallel activities of multiple sRNAs. In a notable example, the regulation of the stationary-phase sigma factor *rpoS* mRNA is subject to positive regulation by three sRNAs (ArcZ, DsrA, and RprA) (43–45) and is negatively regulated by the sRNA OxyS (46), all of which are transcribed in response to different environmental cues and signals. Additionally, ArcZ, DsrA, and RprA display context-specific differences in their RpoS regulatory activity. In nutrient-rich medium, DsrA contributes considerably more to the positive regulation of RpoS than either ArcZ or RprA, whereas in nutrient-restricted medium (with glucose as a carbon source), ArcZ contributes more to the RpoS translation outcome than either DsrA or RprA (43). Broadly speaking, multiple sRNAs can modulate the expression of a single gene but can also function in concert, acting on different targets in related pathways (47) through cross talk (48, 49) or through the action of sponges (50). Furthermore, sRNA regulatory networks can be strongly influenced by the physiological state of the bacterial cell, where global RNA-RNA interaction studies have revealed profound differences depending on the assay medium or stage of growth (51, 52). Some sRNAs also have profound phenotypes in infection models without any such phenotypes *in vitro* (53, 54), suggesting that an appropriate experimental context is critical for observing the effects.

The complexity of sRNA regulatory networks highlights the need for new experimental approaches to systematically probe the functions of sRNAs in bacteria. Due to the association of Hfq with growth defects under nutrient stress (14, 15), we sought to investigate whether deletions of sRNAs would impact E. coli’s growth under conditions of nutrient stress. Herein, we present a high-throughput approach to uncovering cryptic sRNA phenotypes by investigating the growth of sRNA double deletion strains. We characterized the growth of 1,373 double mutants under 32 growth conditions and identified more than a thousand growth phenotypes. In one example, we identified a synthetic lethal interaction between ArcZ and CsrC when E. coli was grown on pyruvate, lactate, oxaloacetate, or d-/l-alanine and provided evidence that this phenotype was a result of the dysregulation of *ppsA*, encoding phosphoenolpyruvate synthase. In all, this work uncovered a densely populated regulatory network of sRNA genes in E. coli that responds to nutrient stress.

## RESULTS

### E. coli Δ*hfq* is impaired for growth on most carbon sources.

**FIG 1:**
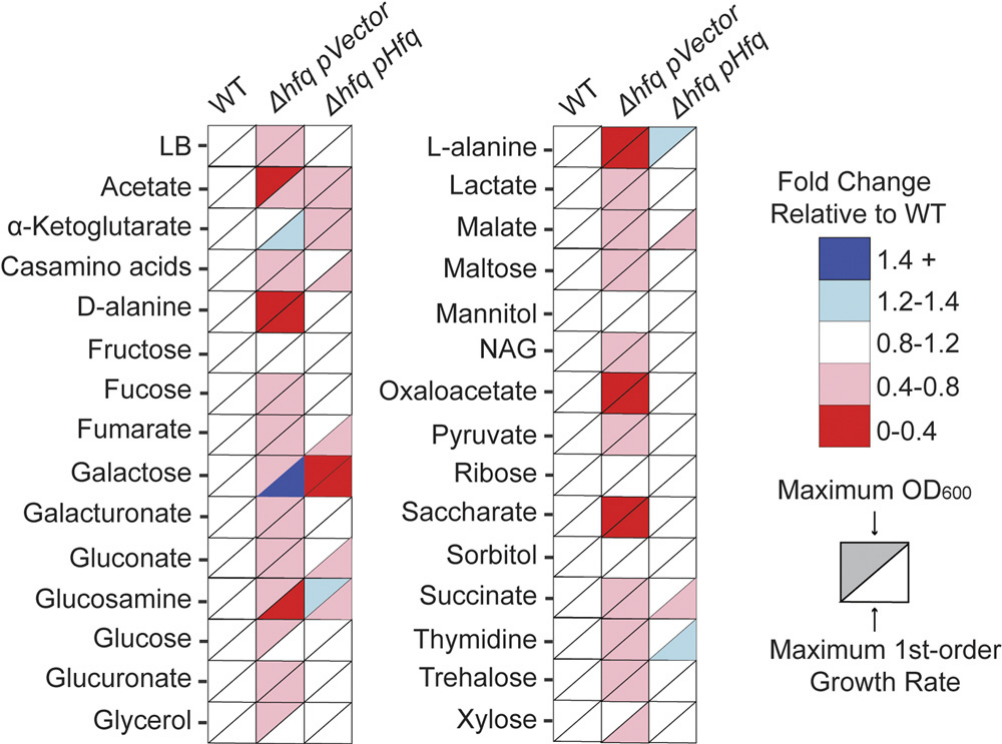
Hfq is important for growth with a variety of different carbon sources. The heatmap shows the growth of E. coli WT and the Δ*hfq* mutant containing an empty vector (pVector) or a high-copy-number Hfq-expressing plasmid (pHfq) in MOPS minimal medium containing the indicated carbon source or in LB. The maximum growth amplitude (OD_600_) and maximum first-order growth rate were calculated from growth curves (*n* = 3) for each carbon source. The average growth for each strain is relative to the growth of WT E. coli under each condition to generate a fold change value that is represented in the heat map. To calculate the maximum first-order growth rate, the first-order derivative of each growth curve was calculated and the maximum for each derivative curve was determined. Plotted is the average (*n* = 3) maximum growth rate for each carbon source.

To assess the impact of a deletion of the sRNA chaperone protein Hfq in different carbon source environments, we measured the growth of wild-type (WT) E. coli and a Δ*hfq* mutant expressing Hfq from a plasmid (28) or carrying an empty vector control in MOPS (morpholinepropanesulfonic acid) minimal medium containing a range of metabolites that can act as sources of carbon to support the growth of E. coli. Specifically, we selected 29 carbon sources that enter the central carbon metabolism at different points and are metabolized by different pathways, as well as a nutrient-rich medium (LB), yielding 30 growth conditions in total. We determined the maximum growth rates and amplitudes (optical density at 600 nm [OD_600_]) in the various media for each of our strains (Fig. 1) and found that the *Δhfq* strain was at least partially impaired for growth under 26 of the 30 conditions tested. This included severe impairment when grown in medium containing acetate, d-alanine, l-alanine, oxaloacetate, or saccharate as a sole carbon source. The expression of Hfq from a plasmid in the Δ*hfq* mutant restored growth to WT levels under almost all conditions (Fig. 1). The presence of the Hfq expression plasmid had a negligible effect on growth across all carbon sources tested (Fig. S1 in the supplemental material).

10.1128/mbio.01225-22.1 FIG S1 The empty vector has minimal effects on E. coli growth. The Δ*hfq* or *Δhfq* mutant containing the vector control was grown kinetically in liquid culture under the different medium conditions outlined in Fig. 1. The maximum growth amplitude (OD_600_) and maximum first-order growth rate were calculated from growth curves (*n* = 3) for each carbon source, and Pearson correlations were calculated to assess the effect of the empty vector on the maximum growth amplitude (i) and first-order growth rate (ii). Download FIG S1, TIF file, 0.9 MB. Copyright © 2022 Rachwalski et al. 2022 Rachwalski et al. https://creativecommons.org/licenses/by/4.0/ This content is distributed under the terms of the Creative Commons Attribution 4.0 International license.

The maximum growth rates of the Δ*hfq* mutant correlated well with the maximum amplitudes (OD_600_) achieved, with the exception of growth in medium containing either galactose or α-ketoglutarate. In these cases, the *Δhfq* mutant had either a slightly reduced or unchanged maximum OD_600_ but a much higher growth rate. While the expression of Hfq from a plasmid restored the growth of the Δ*hfq* mutant to near WT levels under most conditions, it led to severe growth impairment in either galactose or α-ketoglutarate (Fig. S2A). Furthermore, the *Δhfq* mutant entered the logarithmic phase of growth sooner than the WT under these conditions, suggesting that the lack of Hfq enhanced growth. Indeed, we showed that the expression of Hfq in *trans* from the complementation vector led to approximately 8-fold-higher *hfq* mRNA levels and an increased abundance of Hfq protein compared to native expression in the WT strain (Fig. S2B), suggesting that the growth impairment observed in these strains resulted from elevated Hfq expression. Using an inducible Hfq construct (55), we titrated Hfq expression to test whether the growth impairment observed was dependent on Hfq protein levels. We found that increased Hfq protein levels in the cell correlated with the length of the lag phase observed (Fig. S2C). Interestingly, deletion of the Hfq-dependent sRNA Spot42, a known regulator of galactose metabolism (56, 57), did not impact E. coli’s growth on galactose, suggesting that impaired Spot42 activity was not the mechanism of the enhanced growth observed for the Δ*hfq* mutant (Fig. S2D). Furthermore, complementation with binding mutants of Hfq (Fig. S2E) revealed that a mutation in the distal face of Hfq (a change of Y to A at position 25 [Y25A]) resulted in an inability to revert the enhanced growth phenotype, whereas a mutation in the proximal face (K56A) was able to partially revert the enhanced growth phenotype to one similar to that of WT Hfq (Fig. S2E). The distal face of Hfq has been shown to bind AAN repeats of target mRNA species, while the proximal face is important for binding to sRNAs and catalyzing sRNA-mRNA interactions (34, 58), suggesting that this enhanced growth phenotype was a result of abolished mRNA binding to Hfq rather than abolished sRNA activity.

10.1128/mbio.01225-22.2 FIG S2 Hfq at a high copy number impairs growth when E. coli is grown on galactose. (A) Growth of E. coli WT or the Δ*hfq* mutant with an empty vector (pVector) or complementation plasmid (pHfq) in MOPS minimal medium supplemented with galactose was measured over 40 h in a microplate reader (*n* = 3). (B) Hfq expression in the complemented strain was probed by Western blotting (i) and qRT-PCR (ii). For Western blotting, purified Hfq was included as a marker, and subunit α of RNA polymerase was probed as a loading control. For qRT-PCR, RNA was extracted from mid-log-phase cultures (OD_600_ = ~0.5, *n* = 3). Relative expression was determined using *rrsA* (16S rRNA) as a reference gene, and the fold change in expression relative to that of the WT with the empty vector was calculated. (C) (i) Differences in Hfq expression with IPTG-inducible Hfq. Strains were grown to mid-log phase (OD_600_ = ~0.5) in LB medium, and then the expression of Hfq was induced for 30 mins before inoculating into MOPS-galactose medium without IPTG. (ii) Hfq protein levels at time zero of the growth curve were probed using Western blotting. The experiment was performed in biological triplicate, and a representative Western blot from one set of samples is shown. (D) The growth of E. coli WT and the *Δhfq* and *Δspot42* mutants in MOPS minimal medium supplemented with galactose was measured over 40 h in a microplate reader (*n* = 3). (E) The growth of E. coli WT or the Δ*hfq* mutant complemented with a plasmid expressing mutant Hfq protein in MOPS minimal medium supplemented with galactose was measured over 40 h in a microplate reader (*n* = 3). Download FIG S2, TIF file, 2.1 MB. Copyright © 2022 Rachwalski et al. 2022 Rachwalski et al. https://creativecommons.org/licenses/by/4.0/ This content is distributed under the terms of the Creative Commons Attribution 4.0 International license.

In all, we found that E. coli growth on a range of carbon sources was severely impaired by a disruption of *hfq*. This suggested that Hfq and, by extension, one or more Hfq-dependent sRNAs played a critical role in E. coli’s ability to metabolize and grow on different carbon sources. We therefore wondered if phenotypes similar to that of the Δ*hfq* mutant could be uncovered for sRNA deletion strains under these same conditions.

### Fitness of E. coli sRNA deletion strains in different carbon source environments.

To date, 106 sRNAs have been verified experimentally in E. coli, and 44 of these have been shown to bind Hfq *in vivo* (22). We focused our efforts on a subset of known E. coli sRNAs, beginning with a previously described single-gene-deletion collection containing mutants with mutations in 53 sRNA genes along with *hfq*, a *cis-*regulatory RNA (*tisA*), and a transcriptional regulator (*gadE*) (59). A deletion of *gadE* was included as a protein-coding gene for which we did not expect any carbon source-related phenotypes (14). We compared the growth of these strains and that of WT E. coli under 32 different growth conditions. In addition to LB and the nutrient-limited medium supplemented with 29 carbon sources in which we assessed the Δ*hfq* mutant’s growth, we measured the growth of these strains in LB medium with two concentrations of methyl α-d-glucopyranoside (α-MG). α-MG is a chemical inducer of glucose-6-phosphate stress and results in a well characterized growth defect in E. coli *ΔsgrS* (60–63), thus serving as an internal control for our screen.

**FIG 2:**
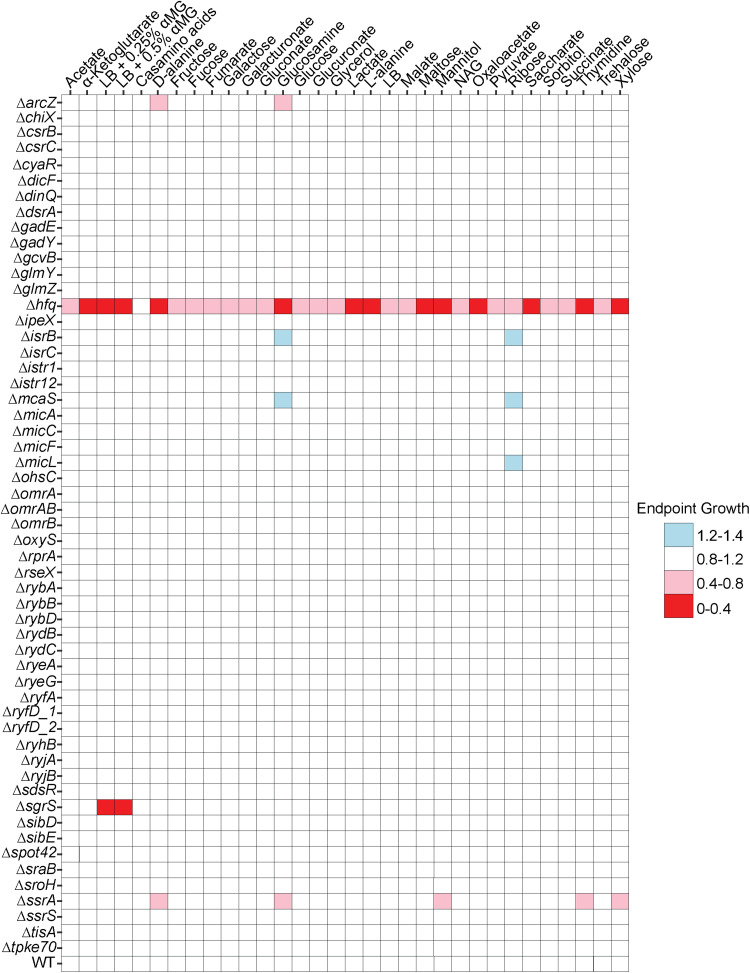
Individual sRNAs are dispensable in E. coli for robust growth on a variety of carbon sources. The heatmap shows the average growth (*n* = 4) of 56 E. coli deletion strains in MOPS minimal medium containing the indicated carbon source or in LB with or without methyl α-d-glucopyranoside. Strains were grown in a 384-colony-density array, and colony size was measured after 24 h and then normalized to the interquartile mean of the remaining strains as described in Materials and Methods.

To facilitate high-throughput analysis of the growth of these strains, we measured colony growth on solid agar medium (14, 27). Four technical replicates of each strain were arrayed on a single agar plate, and transmissive scanning was used to measure colony volumes (integrated densities) for each colony as a proxy for biomass (27). As described above for experiments using liquid medium (Fig. 1), the Δ*hfq* strain was severely impaired for growth on solid agar medium under nearly all nutrient conditions tested (Fig. 2). Notable differences included growth on α-ketoglutarate, mannitol, ribose, and xylose, where the *Δhfq* strain had a severe colony growth defect compared to its growth in liquid medium. Additionally, while the *Δhfq* strain grew poorly in the presence of Casamino Acids in liquid medium (Fig. 1), colony growth was unperturbed. We attributed these differences to altered physiology of E. coli growing in a colony versus in broth. Consistent with previous work (60–63), we observed that both the Δ*hfq* mutant and the *ΔsgrS* mutant were severely impaired for growth in LB supplemented with α-MG. We found additional, modest growth defects for the *ΔarcZ* and *ΔssrA* mutants under several conditions, as well as an unanticipated growth enhancement for the Δ*isrB*, Δ*mcaS*, and Δ*micL* mutants in the presence of glucosamine or ribose (Fig. 2).

One sRNA deletion strain, a Δ*tp2* strain, had a pleiotropic growth defect, comparable only to that of the Δ*hfq* mutant (Fig. S3A). The Tp2 sRNA is expressed from the *pdhR-aceE* intergenic region (64), the entirety of which is replaced with a kanamycin resistance cassette in our *Δtp2* strain (Fig. S3B). However, studies have shown that perturbations of the genes directly downstream from Tp2, *aceE* and *aceF*, result in an inability to grow in fermentative carbon sources, and other studies have suggested that *aceF* could be an essential gene (3, 65). We therefore suspected that the Δ*tp2* phenotypes were a consequence of polar effects on *aceEF* expression. Indeed, similarly to the *Δtp2* strain, the *ΔaceF* strain grows normally in minimal medium supplemented with acetate as a carbon source but is severely growth impaired in minimal medium supplemented with a fermentative (glucose) carbon source (Fig. S3C). Furthermore, expression profiling revealed that *aceE* and *aceF* mRNA levels were significantly reduced in an E. coli *Δtp2* background (Fig. S3D), and we were unable to complement Δ*tp2* phenotypes with the expression of Tp2 in *trans* (data not shown). Accordingly, we excluded *Δtp2* growth data from the heatmap presented (Fig. 2) and removed this strain from further analyses. We suggest that caution be taken in future work when interpreting phenotypes associated with perturbations in this sRNA.

10.1128/mbio.01225-22.3 FIG S3 Deletion of the putative sRNA Tp2 causes severe growth impairment due to polar effects on *aceEF* expression. (A) Heatmap of normalized growth (relative to that of the WT) of the *Δhfq* and *Δtp2* mutants on different carbon sources. (B) Genomic context of WT and Δ*tp2* bacteria at the *pdhR-aceE* intergenic region in E. coli. (C) Liquid medium growth kinetics of E. coli *Δtp2* and E. coli *ΔaceF* in MOPS-acetate (i) and MOPS-glucose (ii). Cultures were grown to the mid-log phase of growth (OD_600_ = ~0.5) in LB, washed, and then used to inoculate either MOPS-acetate or MOPS-glucose medium (1:1,000 dilution). (D) RT-qPCR measuring the expression of *pdhR*, *aceE*, and *aceF* in different genetic backgrounds. RNA was extracted from cultures of the deletion strains grown to mid-log phase (OD_600_ = ~0.5). Relative expression was calculated relative using *rrsA* (16S rRNA) expression as a reference, and then the fold change in expression relative to that of the WT was calculated. Bars show mean values and standard deviations for two biological replicates measured in technical triplicate. Download FIG S3, TIF file, 1.2 MB. Copyright © 2022 Rachwalski et al. 2022 Rachwalski et al. https://creativecommons.org/licenses/by/4.0/ This content is distributed under the terms of the Creative Commons Attribution 4.0 International license.

### Phenotypic profiling of sRNA double deletion strains uncovers novel phenotypes.

**FIG 3:**
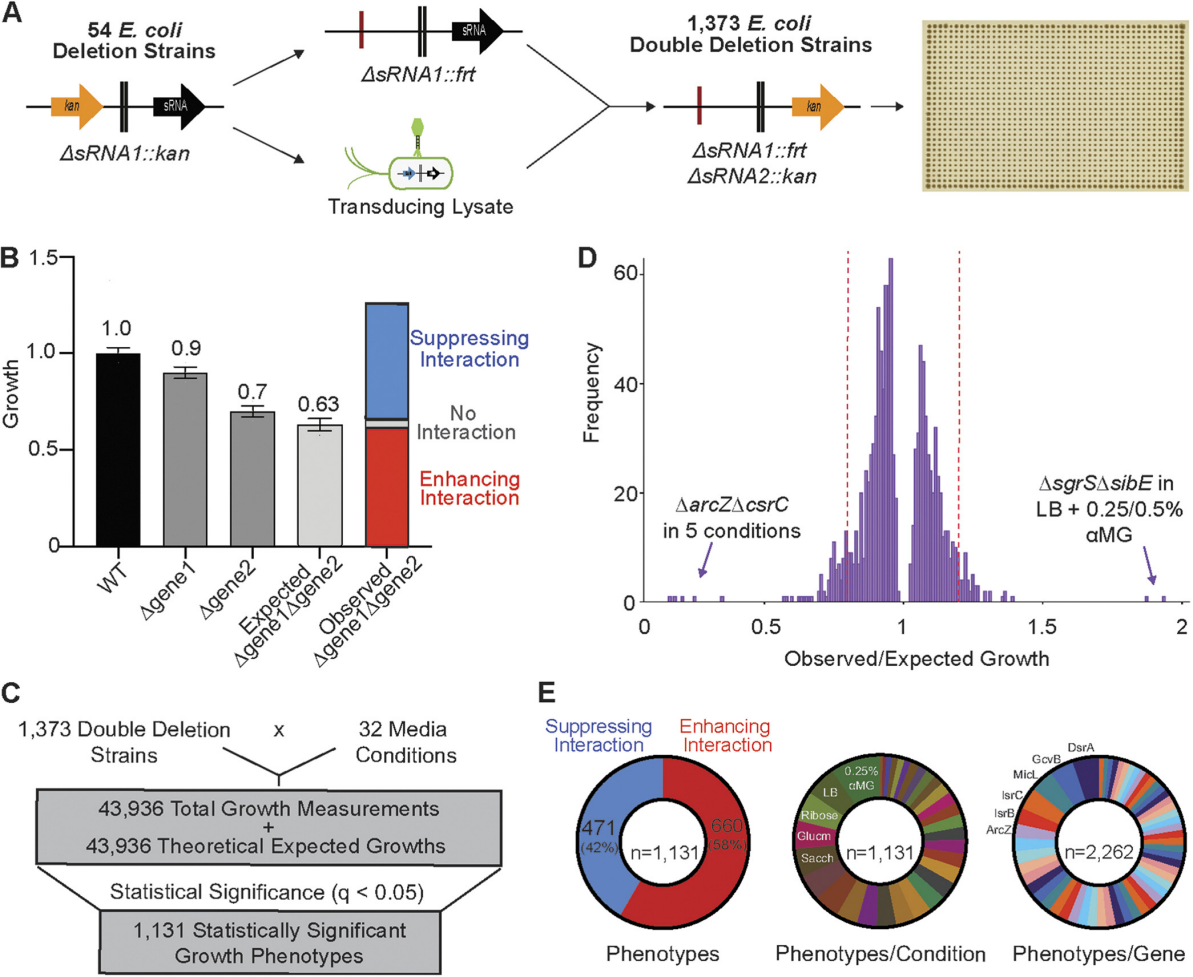
High-density growth arrays uncover context-specific genetic interactions between sRNAs. (A) Schematic outlining an approach to generate E. coli sRNA double deletion strains. Once generated, double deletion strains were arrayed as colonies on solid agar in 1,536-density format. (B) The multiplicative rule was used to calculate synthetic genetic interactions for each strain under each condition. The growth of single and double deletion strains was calculated relative to that of the WT, and the observed growth for a double deletion mutant under each condition was compared to the expected growth, where the latter was calculated as the product of the fractional growths of the single deletion mutants. Where the dominant growth phenotype was growth inhibition, the terms enhancing and suppressing interactions were used to describe phenotypes that were significantly different from the expected growth values. Bars show the mean values and standard errors. (C) In all, 43,936 growth measurements were obtained for double deletion strains. The observed growth of each double deletion strain was compared to the theoretical expected growth (false discovery rate [FDR] ≤ 0.05, Welch’s *t* test), yielding 1,131 statistically significant growth observations. (D) Histogram of fold changes for the 1,131 statistically significant growth phenotypes identified, with dotted red lines denoting a 20% difference in expression from expected growth. Extreme outliers in the data set, the *ΔarcZ ΔcsrC* and *ΔsgrS ΔsibE* mutants, are highlighted. (E) Pie charts summarizing the distribution of phenotypes, with the number of phenotypes per growth condition and total number of phenotypes per gene represented.

In all, we were unable to associate the growth defects of a Δ*hfq* strain with individual sRNA knockouts. While this may have indicated that Δ*hfq* phenotypes were due to sRNAs not represented in our library (for example, many of the newly discovered sRNAs expressed from 3′ untranslated regions [3′-UTRs] of transcripts [66]), we wondered if the lack of growth phenotypes observed could be due to functional redundancies among sRNAs that mask the impact of individual sRNA deletions within our collection. To test the latter hypothesis, we investigated synthetic genetic interactions among these sRNA genes by constructing pairwise deletions of the 54 mutants, creating 1,373 of a possible 1,431 unique double deletion strains using P1 phage transduction (Fig. 3A). After two attempts to generate double deletion mutants using phage transduction, there remained 58 double deletion strains that we were unable to create using this approach. Each of these 58 double mutants was later generated using standard λ-red recombineering (67), indicating that these were not synthetic lethal mutations. Nevertheless, these double mutants were not included in the screening library as they were constructed after data collection.

High-throughput growth experiments were performed on the 1,373 double deletion strains on solid medium containing various carbon sources as described above. These strains were arrayed alongside the 54 sRNA single deletion strains and the WT in a 1,536-colony-density format. To ensure high data quality and reproducibility, we prepared this colony array in two alternate spatial arrangements and screened each configuration in duplicate. We have previously shown that colonies on the edge of high-density arrays have higher growth than those in the middle, although these effects can be mitigated through data normalization protocols (27). These alternate spatial arrangements were an additional measure to validate data normalization, where edge colonies in one arrangement were located centrally in the second configuration (Fig. S4A). Indeed, edge effects were observed in the raw measurements of colony size with both arrangements; however, data normalization corrected the edge effect and resulted in similar normalized growth values for strains regardless of position on the plate (Fig. S4B). Multiple replicates of the double deletion collection were screened under the 32 medium conditions, yielding 43,936 double deletion growth measurements, calculated as the mean of 3 or 4 replicates of each strain under each condition (Fig. S4C).

10.1128/mbio.01225-22.4 FIG S4 Double deletion strain collection was arrayed in two different colony configurations to confirm that normalization methods could eliminate artefacts associated with edge effects. (A) Scanned images of both configurations of the double deletion strain collection array with the locations of some edge colonies highlighted. Highlighted in red are four edge colonies in configuration one along with their new positions in configuration two, and in blue are four edge colonies in configuration two along with their new positions in configuration one. (B) Replicate plots comparing the raw integrated colony densities (i) and the normalized integrated densities (ii) between colony configuration one and colony configuration two when grown in LB. (C) Plot of the calculated normalized growth from all conditions screened. Download FIG S4, TIF file, 2.7 MB. Copyright © 2022 Rachwalski et al. 2022 Rachwalski et al. https://creativecommons.org/licenses/by/4.0/ This content is distributed under the terms of the Creative Commons Attribution 4.0 International license.

When examining growth phenotypes of double deletion strains, genetic interactions were defined using the multiplicative rule (12, 68, 69). Here, a theoretical expected growth value was calculated for each double deletion strain as the product of the growth of the two corresponding single gene deletion mutants (Fig. 3B). We calculated this expected growth value and associated error for each of the 43,936 double deletion growth measurements. The deviation between the calculated expected growth and the empirically observed growth for each double deletion strain under each condition was then assessed for statistical significance using pairwise Welch’s *t* tests, and a Benjamini-Hochberg-corrected *q* value of less than or equal to 0.05 was deemed statistically significant. We determined that 1,131 of the 43,936 double deletion growth measurements (2.57%) showed a statistically significant difference compared to the calculated expected growth (Fig. 3C). Where the phenotype under study was dominantly growth inhibition, we defined genetic interactions that further impaired growth relative to the expected growth as enhancing interactions and those that improved growth as suppressing interactions. A frequency distribution of the magnitudes of these perturbations revealed that the vast majority of phenotypes were subtle (Fig. 3D). Indeed, about 90% of the statistically significant phenotypes uncovered for sRNA double deletion strains affected the expected growth by less than 20%; only 129 interactions—86 enhancers and 43 suppressors—lay outside this range. Interestingly, only two double deletion strains demonstrated an enhancement or suppression phenotype of greater than 50%. E. coli *ΔarcZ ΔcsrC* showed an enhancing phenotype specifically when grown in MOPS supplemented with pyruvate, lactate, oxaloacetate, d-alanine, or l-alanine, and E. coli *ΔsgrS ΔsibE* had a suppressing phenotype in LB with 0.25 or 0.5% α-MG.

Both in the 129 genetic interactions that impacted growth by more 20% and in the broader data set, we observed a higher prevalence of synthetic enhanced growth phenotypes than of suppressed growth phenotypes, at 66% (86/129) and 58% (660/1,131), respectively (Fig. 3E). This observation is consistent with prior synthetic genetic array studies conducted with protein-coding genes, which also show a higher abundance of enhancing genetic interactions (15, 18, 70). From the 660 enhancing interactions identified in this study, just 5 were large, unexpected growth defects (observed/expected growth of <0.4), and all were from the growth of a single double deletion strain under five different conditions: the *ΔarcZ ΔcsrC* strain grown in MOPS minimal medium containing saccharate, oxaloacetate, pyruvate, l-alanine, or d-alanine. Similarly, recent work assessing the growth of ~155,000 double deletion strains with deletions of genes involved in outer membrane assembly revealed approximately 30 enhancing interactions for each of the 39 query genes assayed, but less than 5% of the enhancing interactions were large growth defects (18). Additionally, our average of 42 interactions (median = 36.5) identified for each sRNA represented in the collection aligns with the findings in previous studies (15, 18). The sRNAs with the most interactions in our study were DsrA (118 interactions), GcvB (103 interactions), and MicL (103 interactions), while the sRNAs with the fewest interactions in our study were RprA (18 interactions), RyfD (20 interactions), and RyfA (21 interactions). More than a quarter of all interactions uncovered were found from screening the double deletion collection in LB with 0.25% α-MG (109 interactions), LB (66 interactions), or MOPS medium supplemented with ribose (64 interactions), glucosamine (61 interactions), or saccharate (61 interactions), while the fewest interactions were uncovered in MOPS medium supplemented with mannitol (5 interactions) or l-alanine (8 interactions).

Suppressing interactions were less frequently observed than enhancing interactions; just 471 of the 1,131 growth perturbations (41.6%) led to increased growth relative to the expected growth. Interactions that lead to growth improvement are often overlooked in synthetic genetic interaction studies (15, 18). These interactions occur when the growth impairment of a mutant is relieved by a second suppressing deletion and are strong indicators of antagonistic functional relationships between gene products. In some cases, our suppressing interactions could be explained by a phenotype of growth improvement from a known disrupted function of the corresponding single deletion strain. For example, we observed a suppressing interaction between *ΔarcZ* and *ΔdsrA* mutations in mutants grown on saccharate. ArcZ and DsrA each act independently to activate RpoS expression (43), and the *ΔrpoS* strain has improved growth on saccharate (14). The *ΔarcZ ΔdsrA* double mutation further improved growth, presumably through a mechanism of reduced RpoS expression. Suppressing phenotypes leading to increased growth for the *ΔarcZ ΔdsrA* mutant were also observed when grown on pyruvate, lactate, fucose, and gluconate (Fig. S5), all growth conditions where the *ΔrpoS* strain grew better than the wild type (14).

10.1128/mbio.01225-22.5 FIG S5 Multiplicative-rule bar plots showing the growth of the *ΔarcZ ΔdsrA* strain under 6 different medium conditions, from the double deletion screening dataset. Download FIG S5, TIF file, 1.6 MB. Copyright © 2022 Rachwalski et al. 2022 Rachwalski et al. https://creativecommons.org/licenses/by/4.0/ This content is distributed under the terms of the Creative Commons Attribution 4.0 International license.

**FIG 4:**
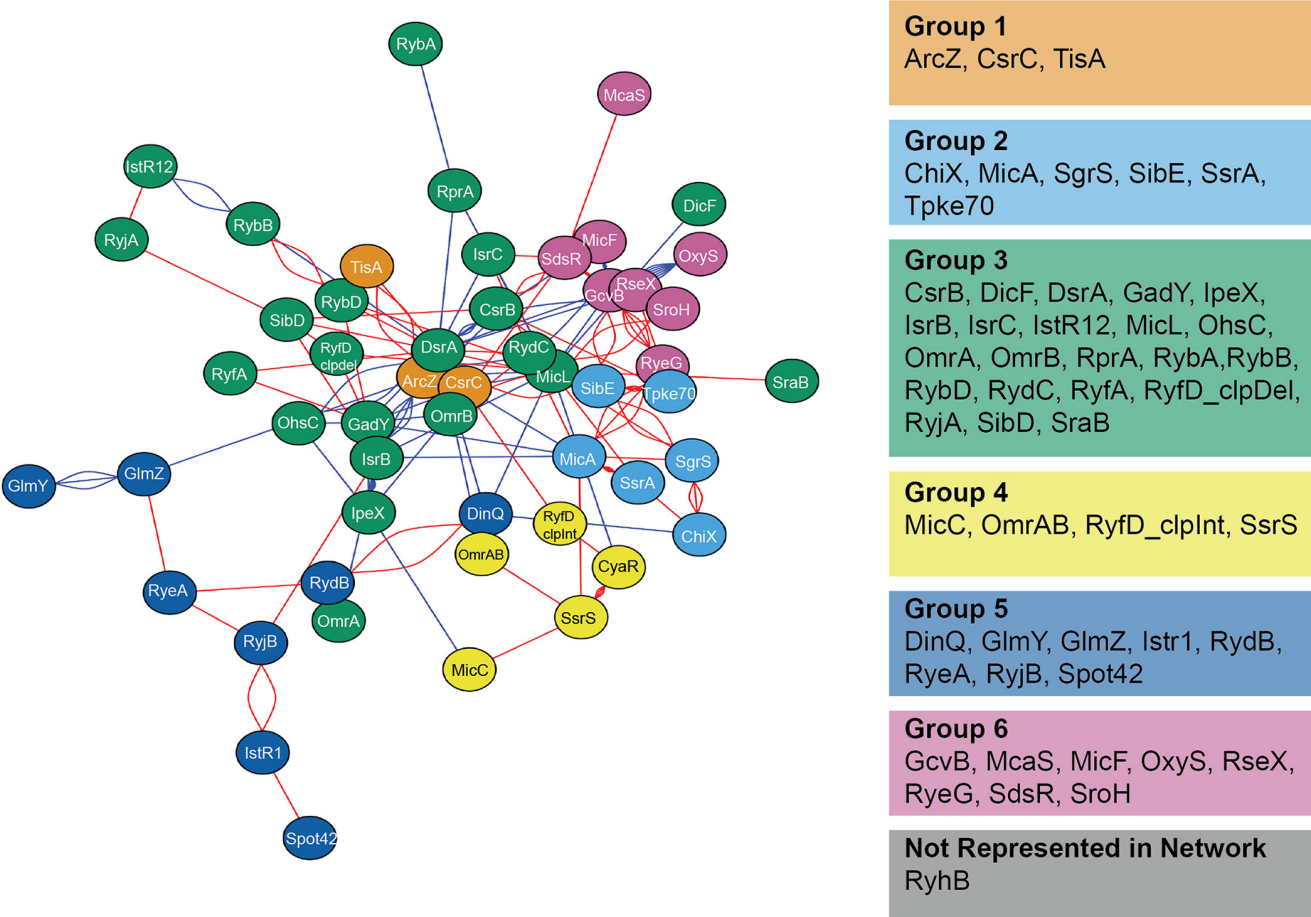
Network map of the 230 statistically significant (*q* < 0.05) growth phenotypes showing a minimum of 15% deviation from expected growth. Interactions between genes are denoted by edges, with each edge representing a double deletion strain grown under a single growth condition where edges are highlighted in either red (synthetic enhancement) or blue (synthetic suppression). The force-directed-network layout was calculated using the Fruchterman-Reingold algorithm, and the Louvain method for community detection was used to cluster sRNAs into groups.

From our data, we generated a phenotypic network map of the 52 E. coli sRNAs included in our study (Fig. 4). Using just the significant interactions in the network map presented in Fig. 4, we employed the Louvain method for community detection (71, 72) to cluster the sRNAs in our study into 6 distinct groups. These groups contain sRNAs that had similar phenotypic profiles in our double deletion study. The network map highlights the dense and complex genetic interactions among sRNAs affecting bacterial cell survival. We created an online browser to maximize the accessibility of our data set and house all the data generated from this study (https://edbrownlab.shinyapps.io/brown_lab_srna_phenobrowser/). Here, users are able to easily access, plot, and download experimental data of E. coli *Δhfq*, sRNA single deletion strains, and sRNA double deletion strains and data from later data sets generated in this study. From the double deletion screen, users can generate index plots of all the growth measurements across all conditions or can investigate the growth of a specific double deletion strain under a particular condition.

### The sRNAs ArcZ and CsrC form a synthetic lethal gene pair in medium supplemented with some carbon sources.

**FIG 5:**
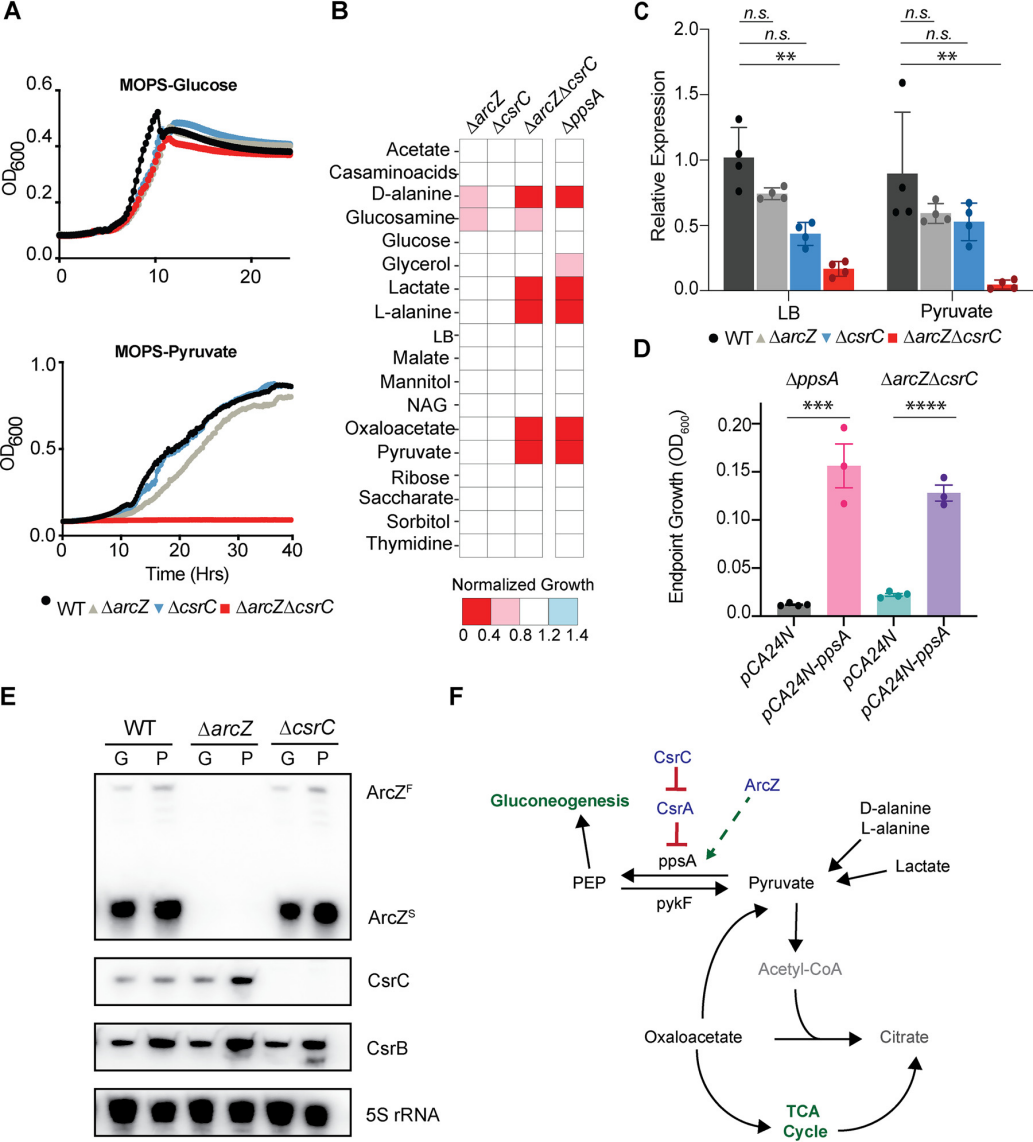
Enhancing interaction for the sRNAs ArcZ and CsrC when E. coli is grown on specific carbon sources. (A) Growth curves of E. coli WT, *ΔarcZ*, Δ*csrC*, and *ΔarcZ ΔcsrC* strains in liquid MOPS minimal medium with glucose or pyruvate as a sole carbon source (*n* = 4). (B) Endpoint growth measurements of *ΔarcZ*, *ΔcsrC*, *ΔarcZ ΔcsrC*, and *ΔppsA* strains grown in liquid MOPS-minimal medium with different carbon sources for 24 h at 37°C with shaking. The cell density (OD_600_) for each strain (*n* = 2) was measured and normalized to the growth of the WT under each condition. (C) Results of qRT-PCR measuring *ppsA* RNA levels in the single and double deletion backgrounds. Cultures were grown to mid-log phase (OD_600_ = ~0.5) in LB, washed, and then resuspended in either fresh LB or MOPS-pyruvate for 30 min before RNA was extracted. Expression relative to the expression in the WT grown in LB was calculated using *rrsA* (16s rRNA) expression as a reference gene. Bars show the mean values and standard errors, and values for individual biological replicates are shown. Groups were analyzed using one-way analysis of variance (ANOVA) with Dunnett’s test for multiple comparisons, *, *P* < 0.05; n.s., not significant. (D) Overexpression of *ppsA* restores the growth defect of E. coli *ΔarcZ ΔcsrC*. Cultures were grown to mid-log phase (OD_600_ = ~0.5) in LB, washed, and used to inoculate MOPS-pyruvate medium (1:1,000 dilution) supplemented with 0.1 μM IPTG, and endpoint growth at 48 h was measured (*n* = 3). Groups were analyzed using an unpaired two-tailed T-test, *, *P* < 0.05. (E) Northern Blot probing the expression of ArcZ, CsrC, and CsrB sRNAs in MOPS supplemented with glucose or pyruvate. Overnight cultures of corresponding strains were grown in MOPS-glucose and then subcultured 1:54 into fresh MOPS-glucose (G) or MOPS-pyruvate (P) medium and grown to mid-log phase (OD_600_ = 0.3, ~4.5 h) before total RNA was extracted. 5S rRNA was probed as a loading control. ArcZ^F^ and ArcZ^S^ denote the full-length and short transcript of ArcZ, respectively. (F) Schematic of proposed regulation of *ppsA* by ArcZ and CsrC.

The most profound genetic enhancements of growth inhibition identified in our study were attributed to one double deletion strain, the *ΔarcZ ΔcsrC* strain, grown in MOPS minimal medium supplemented with l-alanine, d-alanine, pyruvate, lactate, or oxaloacetate. In liquid medium, the growth impairment of the *ΔarcZ ΔcsrC* strain was even more pronounced, as the double deletion strain displayed no growth after 40 h in MOPS-pyruvate, while its growth was unperturbed in MOPS-glucose (Fig. 5A). ArcZ and CsrC both function indirectly to increase the transcription of select genes, through activation of RpoS expression and antagonism of CsrA, respectively (38, 43). As the double-knockout strain was unable to grow on certain carbon sources, we speculated that this phenotype was due to a lack of expression of a direct or indirect target for regulation by both of these sRNAs. Accordingly, we reasoned that a deletion of the target gene should phenocopy the *ΔarcZ ΔcsrC* mutant in those carbon sources. We grew the Keio library, an ordered E. coli genome-scale gene deletion collection of ~3,800 mutants (2), on MOPS-agar medium supplemented with each of the carbon sources glucose, d-alanine, lactate, oxaloacetate, and pyruvate to identify any such target genes. We expected potential target gene deletion mutants to grow unperturbed with glucose but display severe growth impairment with all other carbon sources; 10 gene deletion strains satisfied our primary criteria (Fig. S6A). Due to the strong phenotype observed for the *ΔarcZ ΔcsrC* strain in liquid medium, we selected each of the 10 mutants identified and assessed their growth in liquid MOPS minimal medium with 24 different carbon sources (Fig. S6B). From this experiment, we found that the *ΔppsA* strain best phenocopied the *ΔarcZ ΔcsrC* strain (Fig. 5B). The PpsA protein is a phosphoenolpyruvate synthetase that is required to convert pyruvate into phosphoenolpyruvate, initiating gluconeogenesis when E. coli is grown on pyruvate, lactate, or oxaloacetate (73). The expression of *ppsA* was substantially decreased (>10-fold) in the double deletion strain, particularly after shifting to MOPS-pyruvate medium from LB (Fig. 5C). Interestingly, both *ΔarcZ* and *ΔcsrC* single deletion mutants showed slightly decreased *ppsA* mRNA levels, but not to an extent that resulted in growth impairment on MOPS-pyruvate. This suggested that the decreased *ppsA* level in E. coli *ΔarcZ ΔcsrC* was due to a synergistic effect of the sRNA deletions and that the *ppsA* levels were sufficient for growth in the single deletion backgrounds. Importantly, we found that the expression of *ppsA* from an isopropyl-β-d-thiogalactopyranoside (IPTG)-inducible promoter was sufficient to restore the growth of both E. coli *ΔppsA* and E. coli *ΔarcZ ΔcsrC* in MOPS-pyruvate, confirming that dysregulation of *ppsA* expression is the mechanism of impeded growth in the double deletion strain (Fig. 5D). Additionally, we note that this synthetic lethal growth phenotype is exclusive to CsrC and not CsrB, which acts in a similar manner to antagonize CsrA activity (Fig. S7). We showed that the RNA levels of CsrB and CsrC did not respond equivalently to growth in MOPS-pyruvate (Fig. 5E). Indeed, we saw comparable levels of CsrB induction in all genetic backgrounds when grown with pyruvate, while CsrC was only induced in the *ΔarcZ* strain when grown with pyruvate (Fig. 5E). While not proof positive, neither ArcZ nor CsrC expression was affected in the *ΔcsrC* or *ΔarcZ* strains, respectively, when E. coli was grown in MOPS-glucose, making it unlikely that either of these sRNAs is directly regulated by the other. Rather, we speculate that CsrC is upregulated in MOPS-pyruvate to compensate for the stress associated with a deletion of ArcZ under this condition. We therefore concluded that ArcZ and CsrC likely promote *ppsA* expression in a reinforcing manner when grown in MOPS-pyruvate (Fig. 5F). Further work is required to determine if this is due to direct or indirect regulation.

10.1128/mbio.01225-22.6 FIG S6 (A) The Keio collection was grown in high-density colony arrays on MOPS d-alanine, MOPS lactate, MOPS oxaloacetate, MOPS pyruvate, or MOPS glucose. Deletion strains that were growth impaired (2 standard deviations from the mean) under an experimental condition but not growth impaired in glucose are plotted in the Venn diagram. (B) Phenotypes of the 10 deletion strains identified from the Keio screen were reconfirmed in liquid medium containing different carbon sources. Strains were grown to the mid-log phase of growth (OD_600_ = ~0.5) and then used to inoculate MOPS minimal medium containing different carbon sources in 96-well microplates. Strains were grown for 24 h at 37°C with shaking, and then the cell density (OD_600_) was measured. Shown is the average value from 2 biological replicates normalized to the growth of the WT under each condition. Download FIG S6, TIF file, 1.6 MB. Copyright © 2022 Rachwalski et al. 2022 Rachwalski et al. https://creativecommons.org/licenses/by/4.0/ This content is distributed under the terms of the Creative Commons Attribution 4.0 International license.

10.1128/mbio.01225-22.7 FIG S7 Growth of E. coli WT, *ΔcsrC*, *ΔcsrB*, *ΔcsrB ΔcsrC*, and *ΔarcZ ΔcsrB* strains in LB and MOPS minimal medium supplemented with either glucose or pyruvate was measured over 40 h in a microplate reader (*n* = 4). Error bars shown represent the standard errors of the means. Download FIG S7, TIF file, 0.9 MB. Copyright © 2022 Rachwalski et al. 2022 Rachwalski et al. https://creativecommons.org/licenses/by/4.0/ This content is distributed under the terms of the Creative Commons Attribution 4.0 International license.

### Genetic suppressors may predict targets of sRNA-mediated regulation.

**FIG 6:**
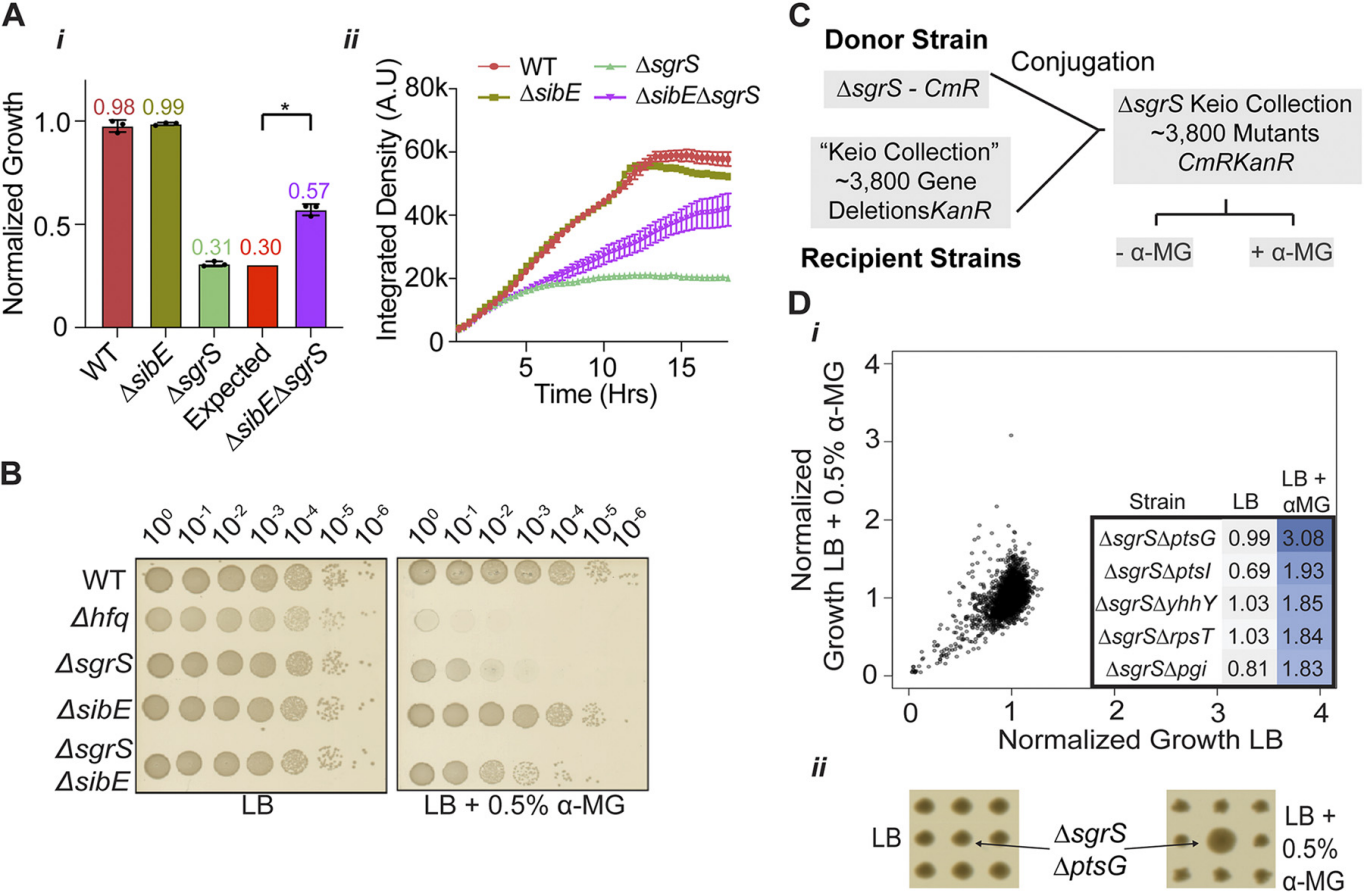
Suppression of α-MG killing identifies possible regulatory targets of SgrS. (A) E. coli *ΔsibE ΔsgrS* has synthetic suppressed growth in the presence of α-MG; endpoint growth (i) and growth over time (ii) from the primary screen are shown. A.U., absorbance units. (B) Serial dilutions from mid-log-phase cultures were spotted on LB agar or LB agar with 0.5% α-MG to reconfirm α-MG suppression phenotype. (C) Schematic showing conjugation of an SgrS deletion into the ~3,800 strains of the Keio nonessential gene deletion collection. (D) (i) The *ΔsgrS* Keio collection was grown on LB agar with and without 0.5% α-MG. Normalized growth of each double deletion strain in LB is plotted against growth in LB with α-MG. The top 5 double deletion strains that antagonize α-MG-mediated growth inhibition are shown in the table to the right. (ii) The strongest suppressor of α-MG killing, the *ΔsgrS ΔptsG* mutation, is highlighted after growth on LB or LB with 0.5% α-MG. Bars show the mean values and standard errors. *, *P* < 0.05.

In many cases, we were unable to rationalize synthetic genetic suppression interactions between sRNA deletions. The strongest of these phenotypes was between *ΔsgrS* and *ΔsibE* when grown in the presence of α-MG (Fig. 6A). While the *ΔsgrS* strain was ~10^5^-fold more sensitive to α-MG than the WT, disruption of *sibE* in this background suppressed α-MG sensitivity >100-fold (Fig. 6B). SgrS is highly induced in response to glucose-phosphate stress, is triggered by α-MG, and promotes bacterial cell survival by limiting the influx of glucose through repression and inactivation of sugar transporters, as well as promoting the conversion of glucose-6-phosphate to glucose through posttranscriptional activation of the YigL sugar phosphatase (61). SibE is a type I antitoxin for the small peptide IbsE, and IbsE inhibits cell growth by depolarizing the inner membrane (74). As IbsE is encoded antisense to SibE, the Δ*sibE* strain is lacking both the SibE sRNA and the IbsE small peptide. To our knowledge, there has been no link found between SibE/IbsE and glucose-phosphate stress to date, which prompted further investigations into the nature of synthetic interactions between SgrS and other genes.

We sought to investigate which deletions of nonessential protein-coding genes from the Keio collection would also antagonize the α-MG-mediated growth impairment of the *ΔsgrS* mutant. High-throughput conjugation was used to introduce an SgrS deletion into each of the mutants of the Keio collection (Fig. 6C). The double deletion strains were then grown in colony arrays on MOPS minimal medium containing either glycerol or LB and with or without 0.5% α-MG. A similar screen was conducted by the Vanderpool group, in which transposon mutagenesis was used to mutagenize a Δ*sgrS* strain, to identify a novel suppressor of α-MG killing, namely, mutation of *pitB* (75). The assay described here represents the first systematic effort to phenotype all nonessential protein-coding genes for α-MG suppression in a *ΔsgrS* background. We identified a number of mutations in protein-coding genes that suppressed the α-MG sensitivity of the *ΔsgrS* strain in LB, the strongest of which was the *ΔptsG* mutation (Fig. 6D). Indeed, a key role of SgrS is to silence PtsG expression and activity in response to glucose-phosphate stress (63, 76), which is thought to limit the influx of glucose while the cell is responding to a build-up of toxic glycolytic intermediates (77). Other strong suppressors of α-MG toxicity in a *ΔsgrS* strain were the *ΔptsI* mutation (PtsI is another component of the glucose phosphotransferase system) and the Δ*pgi* mutation, which would halt the interconversion of glucose-6-phosphate and fructose-6-phosphate (78, 79). Growth inhibition induced by α-MG was much stronger in *ΔsgrS* cells grown on MOPS minimal medium with glycerol than in LB (Fig. S8A). Similar to the case for LB, α-MG toxicity was suppressed by a number of mutations (e.g., *ΔptsG*, *ΔcoaE*, *ΔileS*, *ΔpolA*, and *Δpgi*) in strains grown in minimal medium containing glycerol. Perhaps unexpectedly, we identified several mutations that suppressed the *ΔsgrS* strain’s growth impairment in MOPS-glycerol (e.g., Δ*nlpD*, Δ*aceF*, Δ*yecH*, *ΔrpoS*, *Δcrp*, and *ΔgalM*) that were not detected in experiments in LB containing the same concentration of α-MG (Fig. S8B).

10.1128/mbio.01225-22.8 FIG S8 SgrS cross into the Keio gene deletion collection uncovered suppressors of α-MG activity. (A) Shown are 1,536-colony-density arrays of plate 2 of the *ΔsgrS* Keio collection grown in either LB or MOPS-glycerol with and without 0.5% α-MG. (B) Replicate plot of raw integrated colony density in MOPS-glycerol plotted against raw integrated colony density in MOPS-glycerol with α-MG. The 16 strongest suppressors of α-MG toxicity in MOPS-glycerol are shown in a table to the right of the index plot. Download FIG S8, TIF file, 2.6 MB. Copyright © 2022 Rachwalski et al. 2022 Rachwalski et al. https://creativecommons.org/licenses/by/4.0/ This content is distributed under the terms of the Creative Commons Attribution 4.0 International license.

In all, our synthetic genetic array analysis with the *ΔsgrS* mutant and the Keio collection revealed that targets of SgrS-mediated repression (*ptsG* and *ptsI*) showed the strongest synthetic suppression of the Δ*sgrS* growth impairment under glucose-phosphate stress. Other mutants with lesser effects might alleviate glucose-phosphate stress in a *ΔsgrS* strain by reducing flux through glycolysis and the tricarboxylic acid (TCA) cycle (e.g., *pgi* and *crr*) or through less obvious means (e.g., *yhhY*, *rpsT*, or *nlpD*). These observations led us to speculate that the synthetic suppression observed in the *ΔsgrS ΔsibE* mutant was due to negative regulation of *ibsE* expression by SgrS following sugar-phosphate stress. To that end, we measured SgrS and SibE sRNA levels and *ibsE* mRNA by Northern blotting in three genetic backgrounds (WT, *ΔsgrS*, and *ΔsibE*) following 30 min of α-MG-induced sugar-phosphate stress (Fig. S9). We observed that SibE was subtly induced by α-MG in the WT strain but not in the *ΔsgrS* strain and that *ibsE* levels were elevated in the *ΔsgrS* strain with no further induction of *ibsE* after α-MG stress. The changes in SibE and *ibsE* expression in response to α-MG or the deletion of *sgrS* were modest, and more work is therefore needed to fully elucidate the roles that IbsE and SibE play during glucose-phosphate stress.

10.1128/mbio.01225-22.9 FIG S9 Northern blot probing SgrS, SibE, and IbsE RNA levels in WT, *ΔsgrS*, and *ΔsibE* E. coli with (+) and without (−) α-MG challenge. Overnight cultures of corresponding strains were grown in LB and then were subcultured 1:50 into fresh LB and grown to late-log phase (OD_600_ = 0.8, ~2 h). Cells were then challenged with or without 0.5% α-MG for 30 min, and total RNA was extracted. 5S rRNA was probed as the loading control. Download FIG S9, TIF file, 1.3 MB. Copyright © 2022 Rachwalski et al. 2022 Rachwalski et al. https://creativecommons.org/licenses/by/4.0/ This content is distributed under the terms of the Creative Commons Attribution 4.0 International license.

## DISCUSSION

The molecular functions of most sRNAs have been characterized through transcriptomics approaches followed by biochemical confirmation; however, relatively few growth phenotypes have been found for sRNA deletion strains. Many sRNAs require the chaperone protein Hfq to promote base pairing and stability, resulting in changes in protein synthesis. In contrast to sRNA genes, the deletion of Hfq results in widespread chemical sensitivities and growth defects under a variety of medium conditions (26), as well as attenuated virulence for E. coli, Salmonella enterica, Acinetobacter baumannii, Pseudomonas aeruginosa, and Klebsiella pneumoniae (80–85). Defects in growth observed in the absence of Hfq are often attributed to the loss of function of one or more sRNAs and are used to highlight the importance of sRNAs for bacterial stress response (22). The lack of observed growth phenotypes for sRNA deletion strains, however, prompts an important question: are sRNAs as important to bacterial survival as widely thought if there are few or no consequences for bacterial growth and survival when their activity is disrupted? One explanation for this lack of sRNA growth phenotypes is widespread redundancies in sRNA regulatory networks.

We demonstrated that changes in the available carbon source can drastically alter the fitness of E. coli *Δhfq*. This suggests that Hfq plays an important role in the adaptation to changes in carbon source availability. Indeed, a link between Hfq expression levels and growth rates (86), as well as Hfq and central carbon metabolism, has been observed in E. coli and other *Enterobacteriaceae*. An *hfq* deletion strain of A. baumannii also displayed altered growth in many different carbon environments (87), and transcriptomics studies have identified dysregulation of several genes involved in central carbon metabolism in *hfq* deletion strains of S. enterica (84), P. aeruginosa (88), and K. pneumoniae (85). Where carbon metabolism is strongly linked to bacterial virulence (89, 90), the impact observed herein for the E. coli *Δhfq* strain grown on various carbon sources is certainly consistent with the reduced virulence frequently associated with *hfq* mutation in pathogens (80–84). Due to its role as a major facilitator of sRNA activity in E. coli and related organisms, *hfq* deletion phenotypes have often been attributed to abolished function of Hfq-dependent sRNAs (22). However, despite the extensive *Δhfq* growth phenotypes identified, we were surprised that so few strong growth phenotypes have been reported for sRNA deletion strains (6, 18, 59).

Here, we systematically generated a pairwise collection of 1,373 sRNA double deletion strains, which we then screened for growth phenotypes under 32 different carbon source and nutrient stress conditions using synthetic genetic arrays. The identification of more than 1,000 significant growth phenotypes in this study represents a first and lays a foundation for further synthetic genetic interaction experiments to better understand sRNA functions. A recent screen of the growth phenotypes of E. coli single gene deletion strains in 30 carbon source environments showed that approximately 9% of the nonessential-protein-coding genome (342/3,796) is required for growth under at least one carbon source condition (14). Our phenotype screening of sRNA double deletion strains shows that complex regulation performed by networks of sRNAs is also an important component of the E. coli response to changes in carbon source availability. Next-generation sequencing approaches have been used to define *in vivo* sRNA interactomes by sequencing all sRNA-mRNA chimeras associated with Hfq (41, 51, 52), ProQ (41), and RNase E (91). These studies have identified thousands of interactions between sRNAs and putative mRNA targets and shown that global interactions can differ substantially depending on medium conditions, highlighting the importance of environmental context when characterizing sRNA function. However, systematic sRNA deletion phenotype studies have focused on discovering phenotypes for individual sRNA deletions under a single, often nutrient-rich medium condition (6, 21, 59, 92). By altering the nutrient profile of the medium in which phenotypic testing is conducted, novel sRNA phenotypes can be identified. In one example, large reductions in biofilm formation for E. coli *ΔarcZ*, *ΔdsrA*, and *ΔgadY* strains can be detected when grown in yeast extract-Casamino Acids (YESCA) medium but not in LB (93). Furthermore, phenotypic screening of double deletion strains in synthetic genetic arrays can uncover insights into complex biological networks in bacteria. Synthetic genetic arrays have been used in E. coli to uncover genetic interactions governing nutrient stress (15), cell envelope biogenesis and permeability (18, 19, 94), genome integrity (95), and protein translation (70).

Of note, we identified E. coli *ΔarcZ ΔcsrC* as a conditionally synthetic lethal sRNA pair that rendered a strain with this double mutation unable to grow on specific carbon sources as a consequence of dysregulation of *ppsA*. Both CsrC and ArcZ sRNAs have been indirectly implicated in the regulation of PpsA. CsrC is one of the two sRNAs that negatively regulates CsrA activity at the posttranslational level (38). Transcriptomics studies investigating the CsrA regulon identified PpsA as a target for CsrA-mediated repression (96). Thus, the deletion of CsrC could result in increased CsrA activity, thereby increasing the repression of *ppsA*. Equally, transcriptome sequencing (RNA-seq) studies conducted with ArcZ overexpression identified *ppsA* as a transcript that increases in abundance when ArcZ is overexpressed (97). Additionally, there is evidence of ArcZ binding to the intergenic region between *ppsR* and *ppsA* in an RNA interaction by ligation and sequencing (RIL-seq) study, suggesting that ArcZ is a direct regulator of *ppsA* expression (52). Recently, the small protein encoded by *pssL* has been found to be expressed from the 5′-UTR of *ppsA* and is likely translationally coupled with *ppsA* (98); thus, ArcZ and CsrC are likely to also affect the expression of *pssL*. We demonstrated that *ppsA* transcript levels decreased subtly in both *ΔarcZ* and *ΔcsrC* backgrounds and that *ppsA* expression was severely reduced in the corresponding double deletion strain. Our finding that *ppsA* expression from a heterologous promoter was able to complement the phenotype suggested that the regulatory effects of ArcZ and CsrC were limited to the 5′-UTR of *ppsA*, which was lacking in the inducible *ppsA* expression construct used. We showed that this phenotype was specific to CsrC and not CsrB and that CsrC was considerably more induced in MOPS-pyruvate in a *ΔarcZ* background than in WT E. coli, perhaps as a compensatory response to the absence of ArcZ-mediated regulation. We conclude that the combined effects of deletions in both CsrC and ArcZ sRNAs decrease PpsA levels to an extent where E. coli is no longer able to grow in carbon sources where *ppsA* is required (pyruvate, lactate, oxaloacetate, and d-/l-alanine).

In this study, we have also identified double deletion strains that display improved growth under specific growth conditions. These types of interactions could be explained in instances where both sRNAs regulate a common target, the combined dysregulation of which improves growth. This type of interaction is highlighted in the growth profile of the *ΔarcZ ΔdsrA* strain. Both ArcZ and DsrA are activators of RpoS, and RpoS expression has been shown to decrease drastically in the corresponding double deletion strain (43). Indeed, a *ΔrpoS* strain displays improved growth under many of the same conditions where we observe the *ΔarcZ ΔdsrA* strain to have higher growth (14), suggesting that the phenotypes found are a result of decreased RpoS expression.

In other cases, we have identified interactions contributing to better growth that could potentially predict the regulatory targets of an sRNA. Of particular interest is the sRNA SgrS, which has a well characterized growth defect when grown with α-MG. From our sRNA double deletion screen, we identified the *ΔsibE ΔsgrS* strain as a suppressor of α-MG-mediated growth inhibition. SibE is a type I antitoxin that is found antisense to the IbsE toxin (74) and has not been otherwise associated with α-MG-mediated glucose-phosphate stress. When an *sgrS* deletion mutation was crossed into every strain of the Keio collection, we identified a number of gene deletions that suppressed α-MG-mediated growth inhibition of a *ΔsgrS* strain. The most prominent suppressor of growth inhibition by α-MG was a *ΔsgrS ΔptsG* double mutation. The glucose transporter PtsG is the primary target of SgrS-mediated repression in response to glucose-phosphate stress and is repressed at both the posttranscriptional and posttranslational levels (60, 76). We speculated that, like PtsG, SibE and/or IbsE could be a regulatory target of SgrS. We showed that IbsE RNA levels were subtly higher in the *ΔsgrS* strain and that SibE was no longer induced by α-MG in the absence of SgrS. These observations could partially explain the growth arrest seen upon α-MG challenge in the *ΔsgrS* strain; however, further work is required to fully understand this interaction.

In conclusion, our work highlights the remarkable complexity of noncoding nutrient stress responses that underpin bacterial cell survival. Our approach has identified more than 1,000 growth phenotypes associated with sRNA deletions when E. coli is subject to metabolic stress. Much work is required to understand the molecular mechanisms underlying these findings, and in particular, the precise regulatory interactions that are critical to bacterial survival. Where phenotypes associated with *hfq* disruption are largely unattributable to loss of function in any single sRNA, we likewise envision that the growth phenotypes associated with sRNA double deletions may involve many sRNA-mRNA interactions. Ultimately, our results underscore the strong context dependency of gene essentiality, expanding this view to now include noncoding regulatory RNAs.

## MATERIALS AND METHODS

### Growth conditions, strains, and gene deletions.

Bacteria were routinely cultured in LB at 37°C (10 g/L NaCl, 10 g/L tryptone, 5 g/L yeast extract), supplemented with antibiotics where appropriate (kanamycin, 50 μg/mL; chloramphenicol, 25 μg/mL; and ampicillin, 100 μg/mL) and with 15 g/L agar for experiments on solid medium. MOPS minimal medium (Teknova) was prepared according to the manufacturer’s instructions: components were filter sterilized after preparing liquid growth medium or added to sterile water and agar (1.5% wt/vol) for solid growth medium. Further details about growth media and the 29 carbon sources used in this study are provided in Table S1.

10.1128/mbio.01225-22.10 TABLE S1 Excel sheet containing lists of strains, plasmids, medium conditions, and primers used in this study. Download Table S1, XLSX file, 0.02 MB. Copyright © 2022 Rachwalski et al. 2022 Rachwalski et al. https://creativecommons.org/licenses/by/4.0/ This content is distributed under the terms of the Creative Commons Attribution 4.0 International license.

All strains and plasmids used in this study are listed in Table S1. Escherichia coli BW25113 [*F*^−^ Δ(*araD-araB*)*567 lacZ4787*Δ::*rrnB-3 λ^−^ rph-1* Δ(*rhaD-rhaB*)*568 hsdR514*] was considered WT, and all mutants were constructed in this background. Details on genomic libraries are provided in the relevant sections below.

Mutants were generated by standard methods (67). The collection of single sRNA deletions was created by P1 transduction of 53 kanamycin-resistant sRNA deletion alleles (59) from E. coli MG1655 to E. coli BW25113. Transductants were colony purified twice and confirmed by PCR. Antibiotic-sensitive knockout strains were created by transformation with pCP20 (67) and induction of FLP-recombinase at 37°C overnight; colonies were screened for antibiotic sensitivity and confirmed by PCR. Single colonies of transduced sRNA deletion strains, as well as of the WT and some additional deletion strains (*Δhfq* and *ΔryeG* strains), were used to create a master library plate that was frozen as glycerol stocks in a 96-well microwell plate.

### Generating deletion strains by P1 phage transduction.

P1-transducing lysates were prepared from 1-mL cultures of each of the kanamycin-resistant deletion strains in E. coli MG1655, and P1 phage transduction was performed as previously described (99), with minor modifications. We first used standard methods to transduce the 52 sRNA deletion alleles (59) from E. coli MG1655 to E. coli BW25113 and prepared antibiotic-sensitive single deletion strains by transformation with pCP20 (67). P1-transducing lysates were then used to infect kanamycin-sensitive sRNA deletion strains in a pairwise manner. Each of the possible double deletions was constructed once, with the selection of a transduced kanamycin-resistant allele and recipient strain (kanamycin-sensitive deletion allele) for each pair being essentially random. For medium-throughput P1 transduction, an overnight culture of each recipient strain (kanamycin sensitive) was resuspended in a volume of MC buffer (10 mM MgSO_4_, 5 mM CaCl_2_) equal to the original culture volume, and 100-μL amounts were dispensed into an appropriate number of wells of 2.5-mL 96-well plates (Sigma-Aldrich). Ten-microliter amounts of the appropriate P1-transducing lysate from the P1 lysate stock plate were added with a multichannel pipette, and plates were incubated without shaking at 37°C for 20 min. Sodium citrate (0.1 M, 200 μL) was added along with 500 μL of 2× LB (20 g/L tryptone, 20 g/L NaCl, 10 g/L yeast extract). Plates were sealed with a breathable membrane (AeraSeal; Sigma-Aldrich) and incubated at 37°C with shaking for 2 to 3 h. One-hundred-microliter amounts of each transduction culture were spotted on LB agar plates (6 samples per plate) containing 50 μg/mL kanamycin and 5 mM sodium citrate. Transductants were colony purified on LB agar containing 50 μg/mL kanamycin and 5 mM sodium citrate. Cotransduction of neighboring alleles is possible with P1 phage and could result in reversion of the unmarked deletion mutation to the WT allele (99). The high-throughput nature in which we generated strains did not permit confirmation of all loci in double deletion strains. Instead, double deletions were confirmed by PCR once a phenotype was selected for follow-up experiments. From a genome search, only 7/1,373 double deletion strains in our collection contained sRNAs that were within 20 kb of each other, resulting in reversion being the more likely outcome: these were the *ΔrprA ΔrybB* (5.6 kb apart), *ΔrseX ΔdsrA* (8.4 kb apart), *ΔohsC ΔglmY* (9.3 kb apart), *ΔcsrC Δspot42* (1.1 kb apart), *ΔsibD ΔsibE* (0.3 kb apart), *ΔtisA ΔistR1* (0.3 kb apart), and *ΔtisA Δistr12* (0.3 kb apart) strains.

### Growth kinetics in liquid medium.

Cultures of E. coli were grown overnight in LB medium with selection as required. Cultures were diluted 1:100 into LB without antibiotics and grown at 37°C with aeration at 250 rpm to the mid-log phase of growth (OD_600_ of ~0.5). Cells were then washed three times with sterile phosphate-buffered saline (PBS), diluted 1:5,000 into fresh assay medium (LB or MOPS minimal medium plus carbon source), and added to a 96-well assay plate (Corning). The OD_600_ was monitored for 48 h at 37°C, with shaking, using an Epoch plate reader (BioTek).

### Fitness screening on solid medium.

Strain collections (the Keio collection [2] and sRNA single and double deletion libraries) were stored in 96-well plates as glycerol stocks and pinned onto LB agar using the Singer ROTOR HDA and then grown at 37°C for 18 h. Strain libraries were then upscaled to master plates at the appropriate colony density for screening (384 or 1,536 density). Master plates were used to inoculate freshly prepared assay plates, which were incubated for 18 or 24 h at 37°C to reach endpoint growth (defined as a time period where the growth of a WT strain has plateaued for at least 6 h). Plates were scanned with an Epson Perfection V750 scanner to generate a high-resolution image, and then the integrated density of each colony was extracted from the image using ImageJ (100). Kinetic growth curves on solid medium were conducted by scanning plates every 20 min until endpoint growth using scanners housed within a 37°C incubator (27). Data were normalized as previously described (27).

### Calculating synthetic genetic interactions.

Synthetic genetic interactions of double deletion strains were calculated as previously described (15, 18), with some modifications. The multiplicative rule was used to calculate the expected growth of each double deletion strain under each condition. Here, the expected growth of the double deletion strain under a given condition is calculated as the product of the growth of both single deletion strains under that condition. Growth is defined as the normalized integrated density of the corresponding colonies in the screening array, wherein the calculated expected growth value for the double deletion is the expected colony size of the double deletion mutant. Since both single deletion growth measurements have a corresponding standard deviation (σ), we also calculated the σ value associated with the expected growth value using the propagation of errors formula (101).

Expected growth of ΔaΔb=(growth ofΔa)×(growth ofΔb)

$$\frac{\sigma \left(\text{expected growth of}\Delta a\Delta b\right)}{\left|\text{expected growth of }\Delta a\Delta b\right|}=\sqrt{{\left[\frac{\sigma \left(\text{growth of }\Delta a\right)}{\text{growth of }\Delta a}\right]}^{2}+{\left[\frac{\sigma \left(\text{growth of }\Delta b\right)}{\text{growth of }\Delta b}\right]}^{2}}$$

To identify statistically significant differences between observed growth and the theoretical expected growth, we used Welch’s *t* test and a Benjamini-Hochberg correction for multiple tests. A corrected *P* value of <0.05 was considered statistically significant.

### Synthetic genetic array with the Keio collection.

The Δ*sgrS* allele was introduced into the Keio collection by high-throughput conjugation as previously described (15–18). Briefly, an *hfr*^+^ strain was created by cospotting 10 μL of an Δ*sgrS*::*cm* strain and E. coli BW38244 harboring chromosomal integration plasmid 19 (CIP19) (17) on LB agar with 0.3 mM diaminopimelic acid and incubating overnight at 37°C. Exconjugants were selected on LB agar containing chloramphenicol and spectinomycin. To mate the Δ*sgrS* allele into the Keio collection, 1,536-colony-density arrays of the Keio collection and the *hfr^+^* Δ*sgrS* strain were pinned together onto an LB agar plate containing 0.3 mM diaminopimelic acid and incubated at room temperature for 6 h, followed by overnight incubation at 30°C. Exconjugants were selected by pinning onto LB agar with chloramphenicol and kanamycin. Double mutants were pinned onto LB or MOPS minimal medium with glycerol, with and without 0.5% α-MG, at 1,536-colony density and grown at 37°C for 18 h. Colony growth and synthetic interactions were calculated as described above.

### Western blot assay.

Whole-cell lysates were extracted from cultures in the mid-log phase of growth (OD_600_ of ~0.5) as previously described (28) and were then run on 10% Mini-Protean TGX gels (Bio-Rad). Samples were transferred onto a nitrocellulose membrane using the Trans-Blot Turbo system (Bio-Rad) and were probed with rabbit polyclonal anti-Hfq antibody (102) or monoclonal mouse anti-RNA polymerase antibody (BioLegend) as a control. Membranes were then washed and probed with either IRDye 680RD goat anti-mouse IgG or IRDye 800CW goat anti-rabbit IgG (Li-Cor Biosciences) and were visualized using the ChemiDoc MP imager (Bio-Rad).

### Reverse transcription-quantitative PCR (RT-qPCR).

Total RNA was extracted from cultures grown to the mid-log phase of growth (OD_600_ of ~0.5) using the hot acid phenol method (102), followed by purification with the Monarch total-RNA miniprep kit (New England Biolabs). In brief, 700 μL of each culture was added to hot acid phenol (65°C) with lysis solution (20 mM sodium acetate [NaOAc], 1 mM EDTA, 0.5% SDS) and incubated at 65°C with gentle mixing. Following centrifugation (16,000 × *g*, 10 min, 4°C), the aqueous phase was removed and further extracted 2 more times with 700 μL of phenol chloroform. The final aqueous phase was combined with an equal volume of 100% ethanol and loaded onto the RNA purification column of the Monarch total RNA miniprep kit. From here, RNA purification was conducted according to the kit protocol. RNA (2 μg) was then converted to cDNA using the high-capacity cDNA reverse transcription kit (Applied Biosystems), and cDNA was diluted to 50 ng/μL in TE buffer (50 mM Tris-HCl, pH 8.0, 1 mM EDTA) for storage.

Relative gene expression was determined by RT-qPCR using a CFX96 real-time system (Bio-Rad). Each biological replicate was analyzed in technical triplicate. The reaction mixtures (20 μL) contained 5 ng of cDNA, 500 nM each primer (Table S1), and GB-Amp Sybr green qPCR mix (GeneBioSys). The cycling conditions were as follows: 95°C for 2 min and 40 cycles of 95°C for 15 s and 62°C for 40 s. The relative quantification of target transcripts was calculated according to the cycle threshold (2^−ΔΔ^*^CT^*) method (103) using 16S rRNA (*rrsA*) as the reference gene.

### RNA isolation and Northern blotting.

Total RNA was isolated from log-phase cultures. In the experiment whose results are presented in Fig. S9, overnight cultures were diluted 50-fold in LB and grown to late-log phase (OD_600_ of ~0.8), and then 1 mL of culture was added to 1 mL of fresh medium with α-MG (0.5% [wt/vol] final concentration) where indicated. Cultures were grown for a further 30 min, cells were collected by centrifugation, and RNA was isolated using 1 mL TRIzol (Sigma) according to the manufacturers’ instructions. For the experiment whose results are shown in Fig. 5E, overnight cultures grown in MOPS-glucose medium were diluted 50-fold into 13.5 mL of MOPS-glucose or MOPS-pyruvate and grown to an OD_600_ of ~0.2. One milliliter of ice-cold stop solution (10% acid phenol, 90% ethanol) was added to cultures, which were placed on ice immediately, and then cells were collected by centrifugation. Total RNA was isolated with 1 mL RNAzol RT (Sigma) according to the manufacturer’s instructions. In both experiments, RNA was ethanol precipitated in the presence of 0.3 M sodium acetate and 15 μg of GlycoBlue coprecipitant (ThermoFisher) and resuspended in 20 μL of nuclease-free water.

Four micrograms of total RNA (2 μg per gel) was denatured in load dye (95% [vol/vol] formamide, 0.5× Tris-borate-EDTA [TBE], 3% [wt/vol] xylene cyanol) and resolved on replicate precast 10% polyacrylamide gels containing 7 M urea (Bio-Rad). RNA was transferred to a Hybond-N membrane (Cytiva) in 0.5× TBE using the semidry setting on a Trans-Blot Turbo transfer system (Bio-Rad) and cross-linked by a 1-min exposure to UV light. Membranes were probed twice with 5′-^32^P-labeled oligonucleotides (Table S1) in ULTRAhyb-oligo buffer (Ambion) at 40°C. Membranes were washed 2 times for 30 min with 2× SSC (1× SSC is 0.15 M NaCl plus 0.015 M sodium citrate), 0.1% SDS at 40°C and stripped with boiling 0.1% 0.1× SSC. Northern blots were exposed to a phosphoimager storage screen and imaged with a Typhoon imager (Amersham).

### Data availability.

All fitness screening data generated in this study are available at https://edbrownlab.shinyapps.io/brown_lab_srna_phenobrowser/.
